# The Human TPR Protein TTC4 Is a Putative Hsp90 Co-Chaperone Which Interacts with CDC6 and Shows Alterations in Transformed Cells

**DOI:** 10.1371/journal.pone.0001737

**Published:** 2008-03-05

**Authors:** Gilles Crevel, Dorothy Bennett, Sue Cotterill

**Affiliations:** Department of Basic Medical Sciences, St Georges Hospital Medical School, London, United Kingdom; University of Minnesota, United States of America

## Abstract

**Background:**

The human TTC4 protein is a TPR (tetratricopeptide repeat) motif-containing protein. The gene was originally identified as being localized in a genomic region linked to breast cancer and subsequent studies on melanoma cell lines revealed point mutations in the TTC4 protein that may be associated with the progression of malignant melanoma.

**Methodology/Principle Findings:**

Here we show that TTC4 is a nucleoplasmic protein which interacts with HSP90 and HSP70, and also with the replication protein CDC6. It has significant structural and functional similarities with a previously characterised Drosophila protein Dpit47. We show that TTC4 protein levels are raised in malignant melanoma cell lines compared to melanocytes. We also see increased TTC4 expression in a variety of tumour lines derived from other tissues. In addition we show that TTC4 proteins bearing some of the mutations previously identified from patient samples lose their interaction with the CDC6 protein.

**Conclusions/Significance:**

Based on these results and our previous work with the Drosophila Dpit47 protein we suggest that TTC4 is an HSP90 co-chaperone protein which forms a link between HSP90 chaperone activity and DNA replication. We further suggest that the loss of the interaction with CDC6 or with additional client proteins could provide one route through which TTC4 could influence malignant development of cells.

## Introduction

HSP90 and HSP70 both play a part in the cellular response to stress. However recently it has become apparent that they also co-operate to chaperone a variety of proteins (clients) in multiple cellular pathways in cells which are not stressed. They often play a vital role in the cellular functioning of the client [1]. The precise result of the chaperone interaction seems to vary with the individual client proteins and examples of effects on structure, modification, protein interactions and localisation have all been reported ( reviewed in [2]). HSP90 has also recently gained some prominence as a possible target for cancer therapy in several different types of tumour [3] and a number of phase 1 and 2 trials of anti-HSP90 drugs are currently under way.

The HSP90 co-chaperones are a group of diverse proteins which help HSP90/70 in their action. This group includes other heat shock proteins, TPR containing proteins, cyclophilins and others. The TPR domain is a 34 amino acid protein motif thought to be involved in protein:protein interactions. TPR motif containing proteins are widely distributed across multiple classes of proteins involved in a variety of cellular functions [4] [5] Those interacting with HSP90/70 usually contain 2–3 copies of the motif and the TPR motifs in such proteins are more closely related to each other than to TPR domains from proteins with other cellular functions [6]. It is thought that the TPR motifs are important for direct interaction with the HSP90/70 proteins [7] and precise mapping of the contact points has been achieved for at least one TPR co-chaperone (Hop-[8]). Although the TPR domains provide a way of interacting with the heat shock proteins they do not seem to define a specific protein function. Therefore while some TPR co-chaperones have quite general HSP90/70 related functions (eg Hop-[6]), others have more specific roles associated with particular HSP90 clients or groups of clients (eg Chip [9] Bag-[1] )

We have previously reported the identification of a protein from Drosophila (Dpit47) which shows properties consistent with it being a TPR co-chaperone [10] This protein shows a tight and stoichiometric interaction with DmHsp90, it also shows a tight interaction with DmHsp70, although this is at substoichiometric levels. In addition to DmHsp90/70 interactions Dpit47 associates with a number of proteins which are involved in DNA replication-DNA polymerase alpha, DmCdc6 and DmOrc2/5 ( GC and SC unpublished). All three of these proteins are thought to have a catalytic role in the initiation of DNA replication. In addition cdc6 is also thought to be involved in controlling the co-ordination of DNA synthesis with cell cycle traversal of the G1/S and G2/M boundaries [11] [12] [13] [14] The interaction between Dpit47 and DNA polymerase alpha causes inhibition of polymerase activity. Based on these observations we have proposed that Dpit47 provides a link between DmHsp90 activity and DNA replication and therefore a possible role for DmHsp90 in some aspect of DNA replication.

The TTC4 gene was originally identified through its localisation in a genomic region linked to breast cancer [15])-although no mutations could be detected in the gene in this study. It has also been reported as a pseudogene with a breast cancer association [16]. More recently the gene has been reported to show a variety of mutations–both deletions and point mutations in tissue samples from patients with melanoma [17]. In the Poetsch study related mutations were also reported in three melanoma cell lines, although in a different study of melanoma cell lines no mutations were detectable [18].

In this paper we show that TTC4 is the human orthologue of the Drosophila Dpit47 protein. The two proteins are highly related in sequence, share a common cellular location, and show analogous interactions with both heat shock and the replication initiation protein CDC6. We show that loss of interaction with CDC6 occurs when some of the point mutations identified in melanoma samples are introduced into the TTC4 gene. This suggests a possible route through which TTC4 could affect the development of malignant melanoma. We also present evidence which suggests that TTC4 has a general role in the progression of cancers in addition to melanoma, since the levels of the protein are raised in a variety of tumour cell lines.

## Methods

### Identification and cloning of the TTC4 gene

Identification of the TTC4 gene was carried out using the Blastp programme at the NCBI. The full length gene in pCR2.1 (Invitrogen) vector was a kind gift of J. Cowell (Roswell Park Cancer Institute). All subsequent cloning steps were carried out by amplifying the TTC4 insert using a high fidelity polymerase (Expand–Roche) to introduce appropriate restriction enzyme sites. The gene sequence and cloning sites were subsequently checked by DNA sequencing (Lark Technologies).

The phylogenetic tree was assembled by entering the sequences of the relevant genes into the EMBNET clustal W /phylodendron programmes on line (http://www.es.embnet.org/Doc/phylodendron/clustal-form.html)

### Two hybrid analyses

Human full length cDNA clones for HSP90 protein 1 beta (HSPCB) and HSP70 protein 8 (HSPA8) were obtained form Origene Technologies, Inc. HuCDC6 was obtained from A. Dutta (Dept of Pathology Boston).

TTC4 was cloned in frame in the EcoR1 and Xho sites of the pJG4.5 vector.Hu CDC6 was cloned in frame in the BamH1 and Not1 sites of the pEG202 vector.

HuHSP90 was cloned in frame in the Not1 and Xho1 sites of the pEG202 vector.

HuHSP70 was cloned in frame in the BamH1 and Xho1 sites of the pEG202 vector.

The sequences of all clones were verified by sequencing (Lark Technologies).

Yeast manipulations were carried out using standard two hybrid protocols (Finley lab: Finley R.L.J et al (1997). (http://proteome.wayne.edu/THPL.html)

Quantitative analyses were performed using liquid beta-galactosidase assays as described (http://biochemistry.ucsf.edu/7Eherskowitz/bgal2.html)

### Immunological reagents

#### Manufacture of TTC4 antibodies

Full length TTC4 was subcloned in the pRSETa vector (Invitrogen). The 6Xhis tagged protein was purified under denaturing conditions and the resultant proteins sent to NeoMPS (Strasbourg) for antibody manufacture. Two different rabbit antisera were obtained. The specificity of the antibody was checked by affinity purification of the antibody against 6XhisTTC4 blotted onto Nitrocellulose membrane and competition with GST TTC4. For this GST TTC4 was bound to glutathione sepharose. Undiluted anti TTC4 serum was incubated for 1 hour at room temperature and then briefly spun down to pellet the beads associated with the specific antibodies. The depleted serum was used at a 1/1000 dilution in western blot. For all experiments both antibodies gave the same result.

#### Other antibodies/ immunological reagents

Anti HSP90 (ab13495), anti HSP70 (ab6535) and anti HuCDC6 (ab3220) were from Abcam. Alexa fluor 488 anti mouse and Alexa fluor 594 anti rabbit and Toto3 iodide were from Molecular Probes. Anti alpha-tubulin was from Sigma (clone DM 1A).

### Immunoprecipitation

Extracts for immunoprecipitation were prepared from sub confluent Hela cells. After treatment with trypsin, cells were washed twice with PBS. They were lysed in one volume of PBS, 1% triton X100 and Complete EDTA free protease inhibitors (Roche). After incubation for 10 minutes on ice the lysate was cleared by centrifugation for 5 minutes in a microfuge at 10 000 g at 4°C.

Coupling of antibodies to Protein A sepharose beads and immunoprecipitation was carried out as described in [19]. 50 µl of protein A beads were used for 150 µl of Hela cell extract. Proteins were eluted from the protein A coupled antibody column in a final volume of 40 µl.

### GST pull down experiments

10 µg of GST-TTC4 (wild type or mutant) bound to glutathione beads was incubated with 6 µg of HSP90 in 10 mM Tris pH 7.5, 150 mM NaCl, 0.1% triton X100 and protease inhibitors (complete EDTA free, Roche). After 1 hour at 4°C the pellets were washed in the same buffer, resuspended in SDS PAGE loading buffer and analysed on a 12% PAGE SDS.

### Cell culture/tissue samples

Melanoma cell lines DX3 and LT5.1 were obtained from IR Hart (Barts and the London School of Medicine, London, UK), and grown in RPMI 1640 medium with antibiotics, glutamine, 10% foetal calf serum and 10% CO_2_. Normal human melanocytes (Nohm1 melanocytes [20] and Hermes 3a, an immortalized subline of Nohm1 (([21]?)) were grown in the same medium with tetradecanoyl phorbol acetate (200 nM, cholera toxin (200 pM), human stem cell factor (10 ng/ml) and endothelin 1 (10 nM). Exponential cultures of all other cell types were provided by Yuen-Li Chung (SGHMS).Placental samples (first trimester chorionic villus) were provided by Guy Whitley (SGUL).Heart samples were provided by Nigel Brand (Harefield Hospital).

### Indirect immunofluorescence

Cells were trypsinised and then deposited on poly lysine treated coverslips. Fixation was carried out using 4 % paraformaldehyde diluted in cytoskeleton buffer (1.1 mM Na2HPO4, 0.4 mM KH2PO4, 137 mM NaCl, 5 mM KCl, 2 mM MgCl2, 2 mM EGTA, 5 mM Pipes, 5.5 mM glucose, pH 6.1). The cells were then treated with permeabilisation buffer (PBS, 1% BSA and 0.1% triton X100). The coverslips were incubated with primary antibodies (mouse anti tubulin alpha and rabbit anti TTC4), either for 2 h at room temperature or 4°C overnight. They were washed with permeabilisation buffer and then incubated with secondary antibodies as described above. The secondary antibodies used were Alexa fluor 488 anti mouse and Alexa fluor 594 anti rabbit. The DNA was counterstained with Toto3 iodide. Prior to microscopy the coverslips were mounted in mounting medium Vectashield (Vector).

### Cellular fractionation

This was carried out basically as described [22] Cells were spun down, washed in PBS and lysed by incubation for 30 minutes at 4°C in a hypotonic solution (10 mM tris pH 7.5, 10 mM NaCl, 1.5 mM MgCl2 and Complete EDTA free protease inhibitors (Roche)). Homogenization was carried out in a Dounce homogeniser and cell disruption was checked by phase contrast microscopy. The nuclear pellet was separated from the cytoplasmic fraction by centrifugation at 5,000 g in an Eppendorf bench centrifuge at 4°C. The pellet was then washed twice and resuspended in hypotonic buffer containing 0.75% Triton, and centrifuged at 5,000 g. The supernatant after centrifugation is the nucleoplasmic fraction, and the remaining pellet, was washed twice and resuspended in SDS loading buffer and constituted the chromatin fraction. In each case the final concentration was equivalent to 10000 cells per µl. 5 µl was loaded in each lane. Samples were analysed by SDS- PAGE followed by coomassie staining and western blotting.

### Preparation of extracts from cultured cells for immunoblotting

Cell pellets were washed twice in PBS and then resuspended in SDS-PAGE loading buffer at a concentration of 50,000 cells/µl. The equivalent of 150,000 cells was loaded in each lane. Cell debris was removed by centrifugation before separation by SDS –PAGE.

### Production of TTC4 mutants by site-directed mutagenesis

Point mutations in TTC4 (pJG4.5) were introduced using the Quickchange site directed mutagenesis kit (Stratagene) as described in the manufacturers instructions.

## Results

### Identification of human homologue of Dpit47 as TTC4

**Figure 1:**
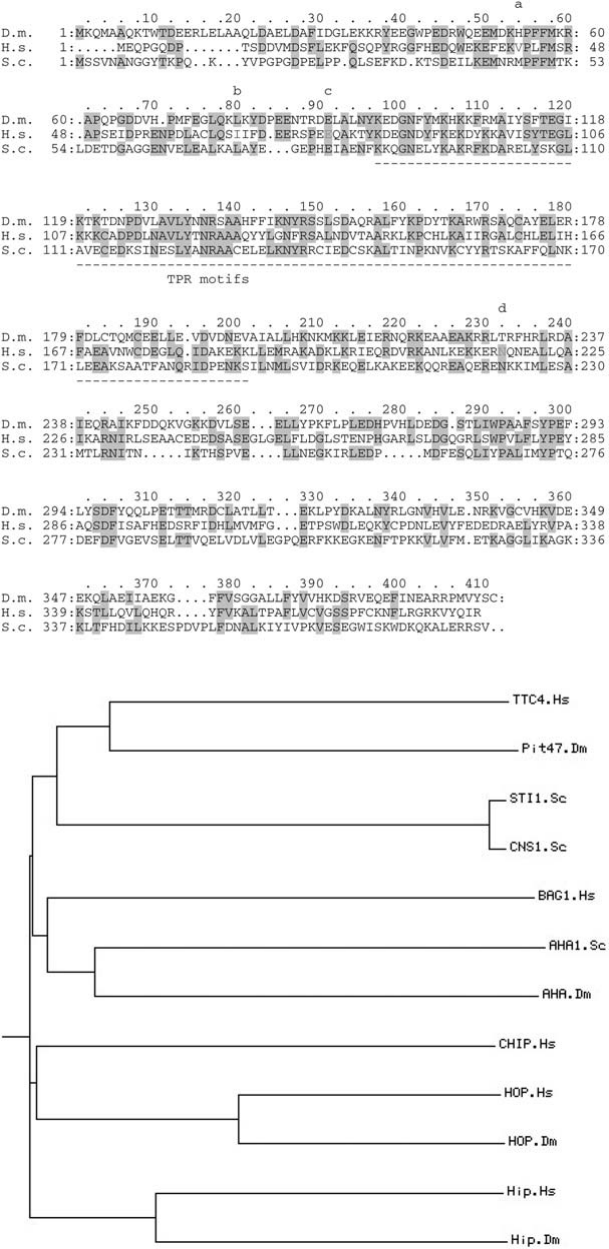
a. Comparison of the protein sequence of humanTTC4 (Hs) with its Drosophila (Dm) and S. Cerevisiae (Sc) orthologues. Areas of identity are shown in shading. The region of the protein containing the TPR domains is underlined. Point mutations of TTC4 which were tested for their interaction with hCDC6 and Hsp90 and 70 (Fig 6) are coloured in red a-42 (V to D), b-67(I to M),c-77 (E to A), and d-217(N to K). b. Phylogenetic tree showing a comparison between the TTC4 protein and a variety of other TPR containing proteins which interact with Hsp90 and/or Hsp70. Each protein is tagged with a prefix to show which species the protein is from h-human, Dm–Drosophila melanogaster, Sc–S. cerevisiae.

The Drosophila Pit47 protein was used to query the NCBI databases in order to find related proteins from other species. The closest homologue from any species was identified as the human TTC4 protein (29% identity). The degree of homology between the two proteins is shown in Fig 1a. Also included in this figure is the CNS1 protein [23] [24]) which is the closest homologue to the Pit47(20% identity) and TTC4 (21% identity) proteins in S.Cerevisiae. All 3 proteins have 3 TPR motifs and the highest homology is concentrated in those regions. However the Drosophila and Human proteins also show homology along the length of the protein particularly in the N-terminal region. Fig1b shows a comparison between TTC4 and a number of other TPR containing proteins from humans and S. Cerevisiae which have been shown to interact with HSP90 and/or HSP70. This further confirms that TTC4 is the most related to the Drosophila Pit47 protein.

### TTC4 interacts with human HSP90 and HSP70

**Figure 2:**
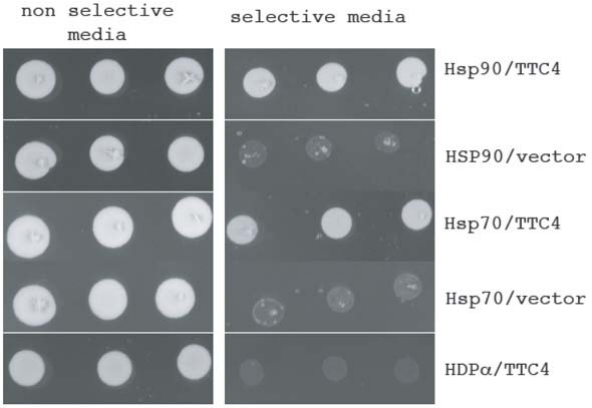
TTC4 shows interaction with human Hsp90 and 70 using the yeast 2-hybrid system. Pairs of bait and activation vectors as indicated were co-transfected into S. cerevisiae and plated as described in Materials and Methods. The growth of each on selective and non-selective media after 1 day is shown.

Our previous studies had shown that the Drosophila Pit47 protein interacts with HSP90 and HSP70. These interactions are very strong and easily detected using two-hybrid analysis. We therefore used similar methodology to determine whether TTC4 showed analogous interactions. The TTC4, human HSP90 and human HSP70 genes were cloned into two hybrid bait and/or reporter plasmids as described in the Materials and Methods. Fig 2 shows that strong growth was seen on non-selective media when the bait was introduced into yeast cells with both test and negative control plasmids (the negative controls used were either the vector alone or the vector containing the Human gene for DNA polymerase alpha large subunit-see also later). However on selective media only the HSP90 and 70 showed significant growth. TTC4 also showed an interaction with Drosophila Hsp90 by two hybrid analysis–although this was only 50% as strong as that between TTC4 and huHSP90 (data not shown).

**Figure 3:**
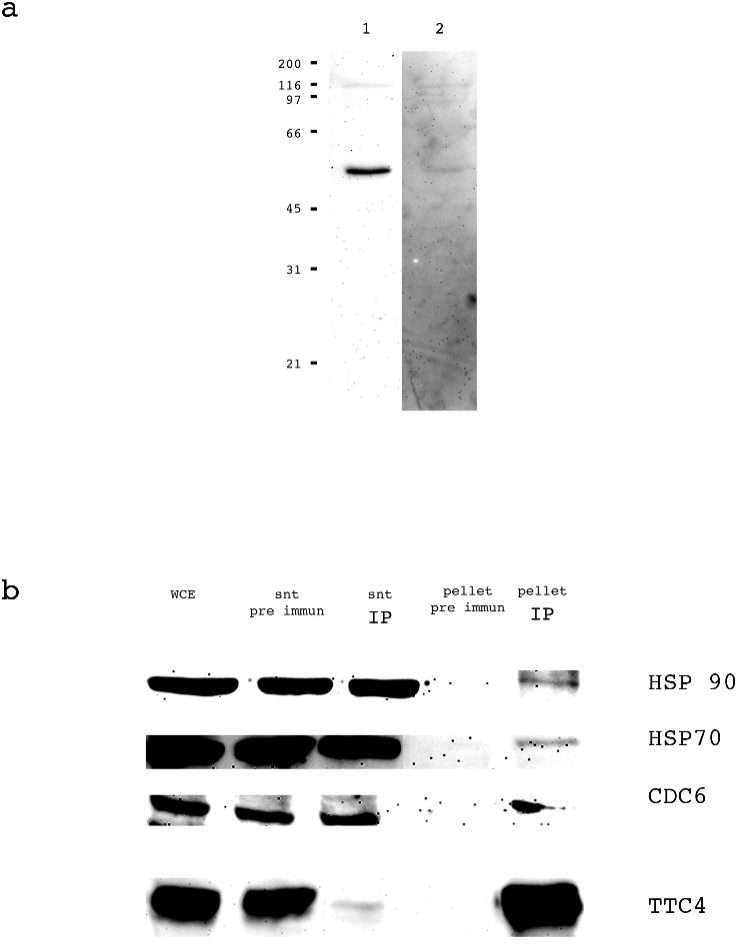
TTC4 shows interaction with human HSP90, HSP70 and CDC6 by immunoprecipitation from Hela cells extract. a: TTC4 serum recognises a single band of 48 kDa in whole cell extracts (lane 1). The intensity of this band is greatly reduced upon competition with GST TTC4 (lane 2). Molecular weight standards are indicated on the left in kDa. b: HSP90, HSP70 and CDC6 co immunoprecipitate with TTC4. The immunoprecipitation was carried out as indicated in Materials and Methods. WCE: Whole cell extract, snt pre immune: supernatant after incubation with the pre immune serum bound to protein A sepharose, snt IP: supernatant after incubation with the anti TTC4 immune serum bound to protein A sepharose, pellet pre immune: eluate from the pre immune serum bound to protein A sepharose, pellet IP eluate from the anti TTC4 immune serum bound to protein A sepharose. For WCE, snt pre immune and snt IP: 40 µg were loaded on the gel. For pellet, pre immune and pellet IP: 1/5 of each eluate was loaded on the gel.

To confirm these interactions we performed immunoprecipitation experiments using anti-TTC4 antibodies. Full length TTC4 protein was overexpressed and used to raise two rabbit antibodies (see Materials and Methods). Both antibodies were seen to react with a band of approximately 48 kDa (estimated molecular weight from sequence data: 44.7 kDa) in extracts (fig 3a lane 1) and behaved similarly in all subsequent analyses. To confirm the specificity of the antibodies GST –TTC4 bound to glutathione sepharose was used to remove specific anti TTC4 antibodies from the serum. When this depleted serum was used on a Western blot the intensity of the 48 kDa band (fig 3a lane 2) was greatly reduced. The anti-TTC4 antibodies were cross-linked to sepharose beads (see Materals and methods) and used to immunoprecipitate TTC4 from Hela cells extracts. TTC4 was efficiently precipitated from the cell extract (fig 3b. compare wce, snt IP and pellet IP). The pre immune serum from the same rabbit was used as a control and was not able to precipitate TTC4 from the cell extract (compare lane wce, snt pre immune and pellet pre immune). The immunoprecipitation pellet was checked for the presence of HSP90 and HSP70 using Hsp-specific antibodies.

Both HSP90 and HSP70 were detected in the anti-TTC4 pellet. Staining of an SDS-PAGE of the immune pellet with colloidal coomassie blue showed that HSP90 was present in near stoichiometric amounts and HSP70 was present at a level of approximately 20% (data not shown). This is similar to what we previously observed for the Drosophila TTC4 homologue [10]. Neither HSP90 and HSP70 were detected in the pre-immune pellet suggesting that the precipitation which we observed was highly specific to the TTC4 antibodies.

### TTC4 interacts with HSP90 via its TPR domain

**Figure 4:**
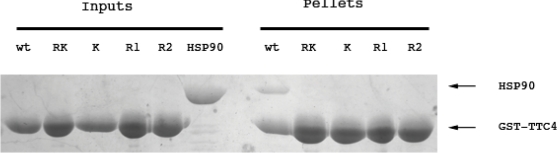
Mutations affecting the TPR domain of TTC4 disrupt binding to Hsp90. The wild type and mutant TTC4 proteins were mixed with HSP90 and incubated as described in Methods. The left panel of this Coomassie stained PAGE shows the purified proteins used in the assay (Inputs) and the right hand panel the glutathione beads resuspended in SDS PAGE loading buffer after co incubation of the proteins (pellets). Wt: wild type GST-TTC4, K: K152E GST-TTC4 mutant, R1 and R2: R156E GST-TTC4 mutant and RK: K152E, R156E GST-TTC4 mutant. More sample was loaded into the lanes with mutant proteins in an effort to detect faint interactions.

A comparison of the TPR sequences of HSP90-binding TPR co-chaperones has suggested that 2 basic residues (Lys-152 and Arg-156 in TTC4) are highly conserved and are involved in the interaction with HSP90 [25]. In order to test whether these residues were also important for the interaction of TTC4 with HSP90 we changed lysine 152 to glutamic acid (K152E) and arginine 156 to glutamic acid (R156E) in GST tagged TTC4. Purified wild type or mutant GST-TTC4 were bound to glutathione sepharose beads and tested for their interaction with purified HSP90. Fig 4 shows that the wild type TTC4 is able to pull down HSP90 efficiently, while both single mutants (K152E and R156E) and the double mutant show no interaction with HSP90. This shows that the TTC4 TPR domain in involved in the interaction with HSP90 and residues previously seen to be needed for the HSP90 interaction of other co-chaperones are important for the interaction.

### TTC4 interacts with HuCDC6

**Figure 5:**
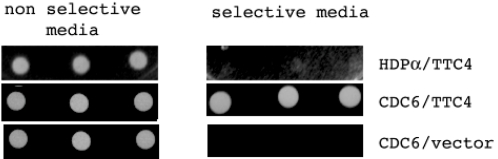
TTC4 shows interaction with human cdc6 using the yeast 2-hybrid system. Pairs of bait and activation vectors as indicated were co-transfected into S. cerevisiae and plated as described in materials and methods. The growth of each on selective and non-selective media after 2 days is shown.

If TTC4 is a true orthologue of Pit47 we would expect that it would show interactions with proteins involved in DNA replication. The Drosophila protein was isolated through a two-hybrid interaction with the large subunit of the DNA polymerase alpha, however when we tried the analogous experiment with TTC4 and the human DNA polymerase alpha we failed to detect an interaction (Fig 2 and 5). We had also however detected interactions (both by two hybrid analysis and immunoprecipitation) of Dpit47 with several other replication proteins including the initiation protein DmCdc6 (GC and SC unpublished). We therefore used 2- hybrid analysis to determine whether TTC4 interacted with the human CDC6 protein. Fig 5 shows that on selective media we detected a strong interaction between TTC4 and HuCDC6, while no interaction was seen when TTC4 was co-transfected with the vector alone or the human DNA polymerase alpha. We also detected an interaction between TTC4 and Drosophila Cdc6 however this was only 50% as strong as that for human CDC6 (data not shown).

This interaction was also confirmed by co-immunoprecipitation (fig 3b) of CDC6 from extracts using TTC4 antibodies but not by immune serum.

### TTC4 is a nuclear protein

**Figure 6:**
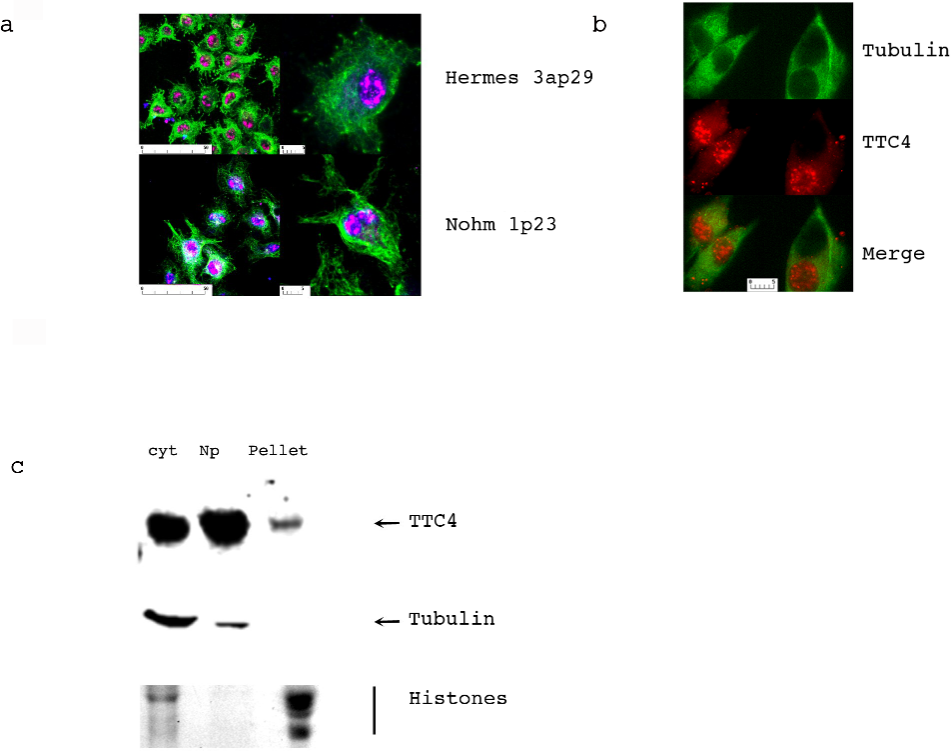
TTC4 is predominantly a nuclear protein. a. Indirect immunofluorescence staining of Hermes 3a and Nohm1 cells was carried out as described in Materials and Methods. In each case TTC4 is stained in red, tubulin in green and DNA in blue. b. Detail of the individual staining patterns of tubulin and TTC4 in Nohm1 cells. c. Immunoblot to show the location of TTC4 after fractionation of Hela cells into cytoplasmic (cyt), nucleoplasm (np) and chromatin associated (pellet) fractions as described in Materials and Methods. Alpha-tubulin (immunoblot) and Histones (Coomassie- blue staining) are shown as controls for the fractionation. Note that the coomassie stained band in the cytoplasmic lane of the histone panel is not histone related.

Dpit47 is a nucleoplasmic protein at all stages of the cell cycle. We therefore analysed the subcellular location of TTC4 to determine if it was comparable. The antibodies were used to check the subcellular localisation of the TTC4 protein using indirect immunofluoresence in the melanocyte lines Nohm 1 and Hermes 3a (Fig 6a/b). In both cases the protein was seen to be nuclear. Nuclear staining was not uniform but had a speckled or clumped appearance. This suggests that there may be some compartmentalisation of the TTC4 protein within the nucleus. No difference was detected between either of these cell lines in terms of location. We also checked the location of TTC4 in two malignant melanoma lines, DX3 and LT5.1. For both of these lines the location of the TTC4 was identical to that in the melanocyte lines (data not shown).

To determine whether TTC4 was tightly bound to chromatin, Hela cells were fractionated into cytoplasmic, nucleoplasmic and pellet fractions (the latter consisting mostly of chromatin and nuclear matrix) as described in Materials and Methods (Fig 6c). The efficiency of the fractionation was checked by detecting the presence of tubulin (western blotting) and histones (coomassie staining) in the different fractions. Tubulin is mainly detected in the cytoplasmic fraction with a small amount in the nucleoplasmic fraction, while histones are only detected in the pellet fraction. This confirms the authenticity of the fractionation. Fig 6c shows that the nucleoplasmic fraction contains significantly more TTC4 than the cytoplasmic fraction or the chromatin-bound fraction. This suggests that TTC4–like Drosophila Pit47- is a nucleoplasmic protein. Similar results were obtained using melanocytes/melanoma cells (data not shown).

### TTC4 is highly expressed in proliferating tissue and tumour lines

**Figure 7:**
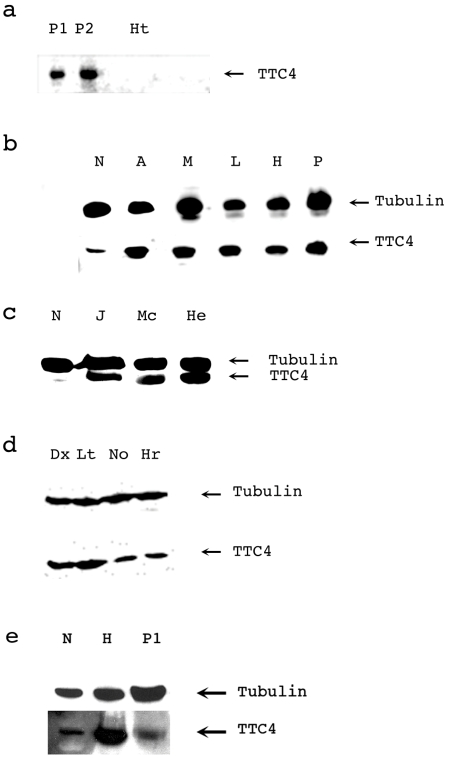
TTC4 expression is higher in rapidly dividing tissue and is also altered in tumour derived cell lines. a. Immunoblot showing the expression of TTC4 in human placental (P1 and P2) and heart (Ht) samples. b and c. Immunoblots showing the expression of TTC4 in a number of tumour derived cell lines. N: normal (MRC5 human lung fibroblast, A: A2780 (ovarian adenocarcinoma), M:MDA (breast), L:Lovo (colon carcinoma), H: HT29 (colon adenocarcinoma), P;PC3 (prostate adenocarcinoma), J: Jurkat (acute t cell leukaemia), Mc: MCF7 ( breast carcinoma) He:Hela (cervical carcinoma). Tubulin loading controls are shown for both blots. d. Immunoblot showing a comparison of the expression of TTC4 in melanocytes (No: Nohm 1 and Hr :Hermes 3a), and malignant melanoma lines (Dx:DX3 and Lt: LT5.1). e. Immunblot comparing the relative levels of TTC4 in placenta and transformed cells (Hela). N: normal (MRC5 human lung fibroblast), H:hela and P1 placenta.

Analysis of the expression of the Drosophila protein at various stages in the life cycle of the fly suggested that the protein was more abundant at those stages where proliferation was taking place. A comparison of the levels of the TTC4 protein in first trimester placental tissue (contains many dividing cells) vs heart (non dividing) (fig 7a) showed that while reasonable levels of TTC4 were visible in two independent placental samples no TTC4 could be detected in an equivalent amount of the heart sample, even at long exposure times. In order to extend this analysis, the TTC4 antibodies were used to probe a human tissue blot (Insta-blot, Imgenex) which contained samples of brain, heart, small intestine, kidney, liver, lung, skeletal muscle, pancreas, spleen, ovary and testis. However none of these tissues contained detectable levels of the TTC4 protein even after extended exposures ( data not shown).

A comparison was also made of the level of the TTC4 protein in normal (MRC5 fibroblasts) versus tumour derived cell lines. Figs 7b, c and d show that in every case there is a significantly more TTC4 in tumour lines than in normal cells. An additional five tumour lines not shown in this figure also showed TTC4 overexpression ( 2fTGH (fibrosarcoma), A549 (lung carcinoma), MG63 (osteosarcoma), Hep2 (epithelial carcinoma), HEC-1b (endometrial carcinoma)). The cells used for this analysis were all from different tissue sources therefore in order to do a more controlled comparison we analysed the levels of the TTC4 protein in two melanoma lines (DX3 and LT5.1) and two melanocyte lines (Nohm1 and Hermes 3a melanocytes). The results from this analysis (fig 7d) also suggested a positive correlation between the levels of TTC4 and the transformation of the cell line. TTC4 therefore shows altered expression in all tumour lines studied but none of the normal cell lines (fibroblasts, Nohm1 and Hermes 3a melanocytes) suggesting that alterations in TTC4 expression may be related to a change in property of the cells.

### The CDC6 interaction is lost in naturally occurring mutants of TTC4

Previous studies have reported a number of abnormalities in the TTC4 gene in melanoma tissues isolated from patients [17] In some cases the changes involve point mutations in the coding region of the gene. To begin an analysis of the functional effects of these mutations, four such reported point mutations were tested to determine whether they might disrupt any of the observed protein interactions of TTC4 (see Fig 1). A two hybrid protocol was used to test the interactions of TTC4 mutated at positions 125 (T to A: codon 42 V to D), 201(T to G: codon 67 I to M), 230 (A to C: codon 77 E to A), and 651(T to A: codon 217 N to K), with the human CDC6, HSP90 and HSP70 proteins. The results from this analysis are summarized in Table 1. None of these mutations had any significant effect on the interactions of TTC4 with the HSP90 and 70 proteins. However two out of the four completely abolished the interaction between TTC4 and CDC6. One of these codons (77) is conserved between Human TTC4 and Drosophila pit47 and mutation of this residue in the Drosophila protein (257 A to C, codon 86 E to A), the interaction with Drosophila cdc6 is also abolished (data not shown).

**Table 1:** Some TTC4 point mutations reported in late stage melanomas lose interaction with CDC6 but still show substantial interaction with Hsp90 and Hsp70.

CDC6/TTC4	100+/−5
CDC6/vector	<5
HSP90/TTC4	100+/−5
HSP70/TTC4	80+/−10
HSP90/vector	<5
HSP70/vector	<5
HDPa/TTC4	<5
CDC6/TTC4-125TA (42VD)	95+/−5
CDC6/TTC4-201TG (67IM)	95+/−5
CDC6/TTC4-230AC (77EA)	<5
CDC6/TTC4-651-TA (217NK)	<5
HSP90/TTC4-125TA (42VD)	80+/−8
HSP90/TTC4-201TG (67IM)	90+/−9
HSP90/TTC4-230AC (77EA)	75+/−10
HSP90/TTC4-651-TA (217NK)	90+/−5
HSP70/TTC4-125TA (42VD)	65+/−12
HSP70/TTC4-201TG (67IM)	70+/−5
HSP70/TTC4-230AC (77EA)	70+/−8
HSP70/TTC4-651-TA (217NK)	70+/−5

Summary of the reactions observed by two-hybrid analysis. All values are expressed as a percentage of the cdc6/TTC4 interaction

## Discussion

In this paper we have presented a number of lines of evidence which suggest that TTC4 is the human orthologue of the Drosophila pit47 protein: 1) The protein sequences of the 2 proteins show high levels of similarity, and this homology is not confined to the region of the TPR motifs; 2) Both proteins are contained in the nucleus and are most likely found mainly in the nucleoplasmic compartment. In each case several putative bipartite like NLS motifs [26] can be seen in the sequence. The localisation for each protein is ‘speckly’ suggesting compartmentalization–although the nature of the compartment is unclear; 3) Both proteins are more abundant in proliferating tissue; 4) Both proteins show strong interactions with the HSP90 and HSP70 proteins. In addition the TTC4:HSP90 interaction needs the same conserved residues that have been seen to be involved in the HSP90 binding of other co chaperones: 5) Both proteins show interaction with the replication initiation protein CDC6. The above observations therefore lead us to suggest that, like Pit47 in Drosophila, TTC4 serves a function linking HSP90 activity and DNA replication in human cells. The role of heat shock proteins in eukaryotes has not been previously investigated although important roles for this class of proteins have been seen in both eukaryotic viruses (eg [27] [28] and prokaryotes [29]. We have seen two notable differences between the proteins. Firstly Dpit47 is upregulated in ovaries [10] whereas TTC4 is not. The significance of this observation remains unclear. In addition the Drosophila protein interacts with the large subunit of the DNA polymerase alpha, however the analogous interaction was not seen for the human protein. This interaction may represent a difference between the two species. However even in Drosophila the CDC6 interaction is significantly stronger than the polymerase interaction, therefore we cannot rule out the possibility that the interaction exists in human cells but cannot be detected because of decreased affinity.

Data presented in this paper also suggests that TTC4 is involved in the development or progression of cancer. A role for TTC4 in melanoma had already been suggested, since mutations seen in the gene in patient-derived samples and cell lines, seemed to correlate with increased invasiveness). Our data suggests that in addition to structural changes in the protein, melanoma lines contain more TTC4 protein than melanocytes. Furthermore increases in TTC4 protein levels are also observed in a number of other tumour derived cell lines compared to normal cells and tissues. It is therefore possible to suggest that an increased level of TTC4 contributes to the development of a variety of different tumours. An increased level of Dpit47 has also been reported in brain tumours in flies [30] A caveat to this argument is that since TTC4 is related to cell stress proteins, it is possible that the increased levels are a response to the abnormal state of the cells. Increases in other stress related proteins have been reported in tumour cell [31]. Although we cannot rule this out, we have seen that the levels of Dpit47 do not increase significantly in response to heat shock (GC and SC unpublished data). Despite the observed correlations between altered TTC4 behaviour and cancer progression we cannot yet define a precise role for the protein in this process. We do not know if the TTC4 protein observed by Western blot represents wild type protein, as TTC4 bearing point mutations similar to those reported for melanomas would not be distinguishable from wild type proteins on polyacrylamide gel electrophoresis. We do not know the function of TTC4 in normal cells. Nor do we know at what stage during the development of the cell lines the amplification has taken place. Such information is necessary in order to propose a meaningful model for TTC4 action during tumour development.

Although the precise role for TTC4 in tumour development is not clear, it may be partly related to a loss of protein interactions. Two of the naturally occurring TTC4 point mutations cause the protein to lose its interaction with hCDC6. Only one of these amino acids is fully conserved in the Drosophila protein but we have observed (GC and SC unpublished results) that mutation of this amino acid in Pit47 destroys its interaction with the Drosophila CDC6. We cannot say that the loss of the interaction with CDC6 is the precise cause of the defect, since the region into which the mutations map is likely to be involved in interactions with a number of proteins. (For instance the same region of pit47 is needed for interaction with DNA polymerase (David Loebel and SC unpublished observation)). In addition interaction of TTC4 with other cellular proteins has also been reported (e.g. TFIIIh [32] and MSL/hampin [33]. However CDC6 is an attractive candidate for this effect. It has a well defined role in the initiation of DNA replication, where it is necessary for the formation of the pre-replication complex. It also has a less well understood role in stalling the progression of cells from the S to M phase of the cell cycle if DNA replication is not complete [11] [12] [13] [14]. Therefore any disruption of its behaviour would be expected to have serious implications for DNA synthesis and the ability of the cell to prevent DNA damage, which could ultimately lead to chromosome instability.
